# On the Use of Water and Methanol with Zeolites for Heat Transfer

**DOI:** 10.1021/acssuschemeng.2c05369

**Published:** 2023-03-08

**Authors:** Rafael M. Madero-Castro, Azahara Luna-Triguero, Andrzej Sławek, José Manuel Vicent-Luna, Sofia Calero

**Affiliations:** † Department of Physical, Chemical, and Natural Systems, Universidad Pablo de Olavide, Ctra. Utrera km. 1, ES-41013 Seville, Spain; ‡ Energy Technology, Department of Mechanical Engineering, Eindhoven University of Technology, P.O. Box 513, 5600 MB Eindhoven, The Netherlands; § Eindhoven Institute for Renewable Energy Systems (EIRES), Eindhoven University of Technology, P.O. Box 513, Eindhoven 5600 MB, The Netherlands; ∥ Academic Centre for Materials and Nanotechnology, AGH University of Science and Technology, Kawiory 30, 30-055 Kraków, Poland; ⊥ Faculty of Chemistry, Jagiellonian University, Gronostajowa 2, 30-387 Kraków, Poland; # Materials Simulation and Modelling, Department of Applied Physics, Eindhoven University of Technology, P.O. Box 513, 5600MB Eindhoven, The Netherlands

**Keywords:** hydrophilic and hydrophobic zeolites, methanol and water adsorption, heat storage, Dubinin−Polanyi theory, storage density

## Abstract

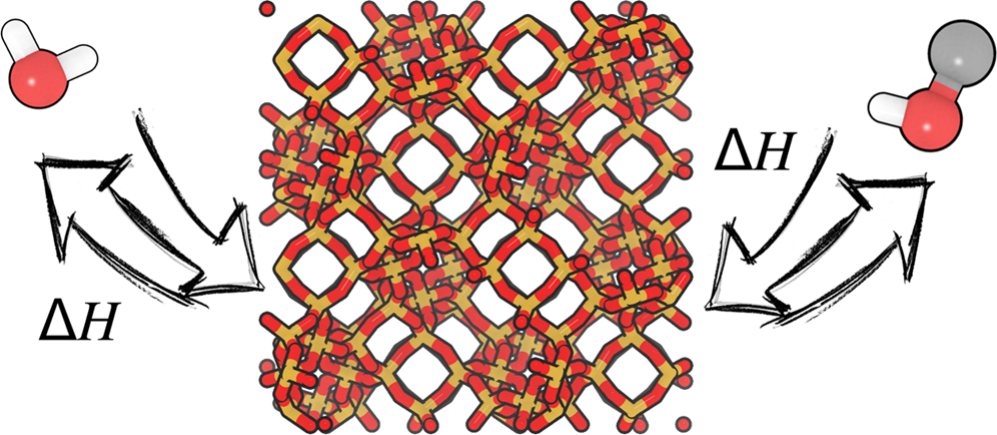

Reducing carbon dioxide emissions has become a must in society, making it crucial to find alternatives to supply the energy demand. Adsorption-based cooling and heating technologies are receiving attention for thermal energy storage applications. In this paper, we study the adsorption of polar working fluids in hydrophobic and hydrophilic zeolites by means of experimental quasi-equilibrated temperature-programmed desorption and adsorption combined with Monte Carlo simulations. We measured and computed water and methanol adsorption isobars in high-silica HS-FAU, NaY, and NaX zeolites. We use the experimental adsorption isobars to develop a set of parameters to model the interaction between methanol and the zeolite and cations. Once we have the adsorption of these polar molecules, we use a mathematical model based on the adsorption potential theory of Dubinin–Polanyi to assess the performance of the adsorbate-working fluids for heat storage applications. We found that molecular simulations are an excellent tool for investigating energy storage applications since we can reproduce, complement, and extend experimental observations. Our results highlight the importance of controlling the hydrophilic/hydrophobic nature of the zeolites by changing the Al content to maximize the working conditions of the heat storage device.

## Abstract 2

Adsorption/desorption cycles of water and methanol in zeolites can be efficiently utilized for thermal energy storage applications to supply the energy demand.

## Introduction

A considerable decrease in energy consumption is essential for the mitigation of global warming.^1−3^ The use of renewable energies, along with the reduction of fossil fuels, is important here.^4,5^ To mitigate this problem, solar, wind energy, or biofuels are promising candidates, but the intermittent nature of renewable energies limits their application for social consumption. This is motivating researchers to work on new approaches for the storage of renewable energy.^6−8^

There are many methods for energy storage at the industrial level based on converting renewable energy into potential energy. One of the most used is pumped-storage hydroelectricity (PSH) or pumped hydro energy storage (PHES). PSH uses the surplus energy obtained in hydroelectric dams in low power demand hours to elevate water from lower to higher levels. That converts the surplus energy into potential energy, which can be used in high power demand periods.^9−15^ However, hydroelectric dams have an impact on the environment.^16^ Another method is compressed air energy storage (CAES).^6,17−20^ CAES uses renewable energies, mainly wind, to compress air at high pressure and generate electricity.^21^ Based on the same principle of stored mechanical energy, flywheel energy storage (FES) uses inertia for storage.^22^ The operation of the system consists of a rotor that is driven and keeps spinning to store kinetic energy.^19,23−26^ The best-known storage method is electrochemical storage, specifically lithium-ion batteries.^27−30^ The current expansion of cell phones or hybrid cars^31^ has increased the need for developing the technological market in this field.^32^ The main limitations of batteries are the loss of capacity^33^ and the risk of thermal runaways or explosions.^29^

In the context of finding alternative methods, thermal energy storage (TES) in three variants (using sensible, latent, or thermochemical heat) has been proposed.^8^ Adsorption-based energy storage belongs to thermochemical heat storage. This technology is based on the adsorption of (gas–liquid) adsorbates with solid adsorbents, storing energy in the desorption phase (charging) and releasing energy in the adsorption phase (discharging). Thus, the efficiency of the heat storage process strongly depends on the adsorbent–adsorbate interactions. Porous materials, such as metal–organic frameworks (MOFs),^34−36^ silica gels,^37−39^ activated carbons,^40−42^ or zeolites,^43−46^ have proven to be promising candidates for this application. The most common adsorbates are water,^47,48^ ammonia,^34,49^ and light alcohols,^50,51^ water being the most studied molecule for storing energy in diverse porous materials.^51−53^

Because adsorption-based energy storage is a promising alternative, the number of experimental and simulation studies is rising. In this context, numerical modeling and molecular simulations are excellent tools to complement experiments. Tatsidjodoung et al.^44^ studied the water–NaX zeolite pair to store thermal energy from the sun. This work concluded that, although there are slight discrepancies between experiments and numerical calculations, simulation is an excellent method for making feasible predictions. Semprini et al.^52^ studied the energy transfer between the 13XBF zeolite and water and its orientation toward the construction of refrigerants, finding a good agreement between simulations and experiments. Lehmann et al.^53^ studied the influence of the cation (sodium or calcium) in zeolite X and water working pairs for energy storage applications. They revealed the importance of working conditions, such as vapor pressure, in the thermochemical energy properties, such as the energy storage density or simply the storage density (SD), which is defined by the quantity of heat which can be stored in a unit mass or volume of adsorbent. Similarly, Kohler et al.^51^ studied the energy stored in zeolite NaX using water as a working fluid, showing the influence of the desorption temperature in the storage density. They compared their values with the energy stored by activated carbons with alcohols as working fluids and noted that the adsorption capacity is as important as the interactions with the adsorbent. Stach et al.^54^ studied the influence of Na and Mg cations and their ratio in zeolites and silica gels using water. Most studies in the literature involve NaX zeolite (with FAU topology), as it is one of the most popular commercial zeolites. It is worth mentioning that NaX usually operates at very high desorption temperatures, typically over 500 K. This is due to the high hydrophilicity of the structure caused by the content of sodium cations. However, other FAU-type zeolites are proposed as interesting alternatives. Ristić et al.^48^ highlighted the significance of decreasing the desorption temperature to optimize the low-temperature heat storage density. To this end, they used NaY zeolite, which is equivalent to NaX but with a slightly higher Si/Al ratio, to study the adsorption heat storage with water as the working fluid. To reduce the desorption temperature of the water, they proposed six post-synthesis-modified samples from the chemical treatment of NaY. The modified adsorbents reduced the desorption temperature up to 30 K, showing a maximum performance at temperatures ca. 400 K. This is an improvement compared to the operating conditions of the NaX zeolite, as discussed above. However, for low-temperature applications, a working pair that lowers the desorption temperature near room conditions is preferred. In this regard, we propose to regulate the hydrophilic degree of the adsorbent by controlling the Si/Al ratio of the zeolite. This way, the performance of a low-temperature process can be maximized while avoiding the post-synthesis treatment step, which may reduce production costs.

This work combines experimental techniques, molecular simulation, and thermodynamical and mathematical modeling for the study of water and methanol adsorption-based energy storage in FAU-type zeolites (FAU)^55,56^ with different Si/Al ratios.^57^ We have chosen methanol as an alternative to water as the conventional working fluid. Our previous publication investigated the adsorption-driven heat transfer of the four first aliphatic alcohols for heat storage applications using activated carbons.^58^ We concluded that methanol exhibits the best performance among the other alcohols for heat storage applications in large pore nanoporous materials. We analyzed the effect of the hydrophobic/hydrophilic nature of the adsorbent on the adsorption behavior, external operating conditions, and energy storage. We used quasi-equilibrated temperature-programmed desorption and adsorption (QE-TPDA) experiments to measure the adsorption isobars of the working pairs. Molecular simulation was used to shed light on the adsorption mechanism from the atomistic level. To this aim, we developed a set of Lennard-Jones parameters that define the FAU-methanol interactions independently of the ratio of cations in high-silica (HS) FAU, NaY, and NaX structures. Finally, we used a thermodynamical model to correlate the adsorption properties with the energy storage of each particular working fluid-zeolite pair.

## Methodology

### Experimental Details

Three samples of FAU were used for the adsorption experiments. HS-FAU is Na^+^ exchanged dealuminated high-silica faujasite with Si/Al > 100, NaY is Na^+^ exchanged faujasite with Si/Al ≈ 2.61, while NaX is Na^+^ exchanged faujasite with Si/Al ≈ 1.06. The characteristics of these materials, i.e., low-temperature nitrogen adsorption and powder X-ray diffraction, were reported in our previous works.^59,60^

Adsorption measurements were performed using the quasi-equilibrated temperature-programmed desorption and adsorption (QE-TPDA) technique. This instrument is a homemade modified setup for temperature-programmed desorption (TPD), which was described in detail in previous works.^61,62^ The samples of 7–10 mg were placed in a quartz tube and activated by heating in He flow (6.75 cm^3^ min^–1^) up to 400 °C (HS-FAU, NaY) or 500 °C (NaX) with a 10 °C min^–1^ ramp and cooling it down to RT. Adsorption was measured in a flow of He containing water steam (saturated) or methanol vapors (*p*/*p*_0_ < 0.05). The samples were heated to induce desorption and cooled to induce adsorption. Each profile was averaged over three desorption–adsorption cycles. For methanol, we used a 4 °C min^–1^ ramp for all materials, while for water, 2 °C min^–1^ for NaY and NaX and 1 °C min^–1^ for HS-FAU were used. It is worth mentioning that adsorption measurements of water in NaX and HS-FAU were reported in our previous work.^59,60^ Between each cycle, they were kept at RT for 2 h. More details on data reduction and methodology are available in the literature.^63^

### Simulation Details

We carried out Monte Carlo simulation in the grand canonical ensemble (GCMC) to obtain the adsorption properties of water and methanol in the three selected zeolites. We performed a minimum of 5 × 10^5^ MC cycles to ensure the adsorption data is fluctuating around equilibrium values. After the equilibration procedure, we conducted additional 2 × 10^5^ cycles for the final production runs. All simulations were performed using RASPA simulation software.^64^ Adsorbent–adsorbate, adsorbate–adsorbate, and adsorbate–cation interactions were defined with van der Waals and electrostatic interactions via the Lennard-Jones and Coulombic potentials, respectively, while we used a Coulombic potential to model the adsorbent–cation interaction. We truncated the potential with an effective cutoff of 12 Å, and we used the Ewald summation method^65^ to compute the long-range electrostatic interactions.

The adsorbents are zeolites with FAU topology; NaX, NaY, and HS-FAU with Si/Al ratios of 1.06, 2.61, and 100, respectively. These structures contain 88, 56, and 2 Al in the unit cell, respectively, and the same number of Na cations to compensate for the net negative charge of the system. The structural models were reported in previous works,^59,60,66,67^ which were created following the methodology developed by Balestra et al.:^68^ (i) random distribution of Si atoms following Lowenstein’s rules, (ii) extra-framework cations initially located at their crystallographic positions, and (iii) structural minimization using Baker’s^69^ method with full-flexible core–shell potential.^70,71^ More details about the assembly of the structures can be found in the Supporting Information (Section S1).

To describe the molecules of water, we used the flexible SPC/E model,^72,73^ and for methanol, the TraPPE model.^74^ Force fields to model the water and methanol adsorption curves in zeolites can be found in the literature.^75−78^ Xiong^76^ studied the interaction between molecules of water and alcohol with pure silica-type MFI-zeolite but without extra-framework cations in the system. Di Lella et al.^75^ provided a set of parameters to reproduce water adsorption in FAU-topology zeolites. However, the parameters and charges of each zeolite–water pair are dependent on the composition of the adsorbent, making this set highly specific and nontransferable. In this work, we used a transferable set of Lennard-Jones parameters and zeolite and cation point charges^79^ that are independent of the Si/Al ratio. Specific Lennard-Jones parameters for the pair interactions for water-zeolite were taken from our previous work,^60^ while for methanol-zeolite, they were unavailable. To sort this out, we parameterized these host–guest interactions by fitting to the experimental adsorption isobars measured with QE-TPDA experiments. Additional details about the parameterization procedure and final set of parameters can be found in the Supporting Information (Section S2 and Tables S1−S3). For the crossed interactions, we used Lorentz-Berthelot mixing rules.^80^ The sodalites cages in FAU zeolites, accessible to water molecules, were artificially blocked for the molecules of methanol.

### Mathematical Model

QE-TPDA experiments and GCMC simulations provided the adsorption properties of zeolites–fluids working pairs. Using these results and a mathematical model based on the adsorption theory of Dubinin–Polanyi,^81^ we predicted the adsorption-based energy storage. This theory is based on the idea that the adsorption mechanisms in micropores is due to the volume filling by the gas molecules instead of surface coverage by successive adsorbed layers.^54^ A brief description of the thermodynamical model as used in this work can be found in the Supporting Information and is also available in the literature.^43,48,51,53,54,58^ In short, we first used the adsorption isobars and isotherms to calculate the adsorption characteristic curve.^82^ This reduces the two-dimensional relation between loading (*q*(*p*,*T*)), temperature (*T*), and pressure (*p*), to the temperature–pressure invariant characteristic curve (*W*(*A*)). This means that adsorption isotherms/isobars at different conditions should fall into the same characteristic curve.^43,58^ This property makes the characteristic curve a useful tool to predict adsorption values at different working conditions. Figure S2 shows a comparison of adsorption isotherms of water and methanol in the three zeolites at different temperatures obtained from GCMC simulations with those predicted from the characteristic curve. The agreement between computed and predicted adsorption properties is a requirement for the application of this theory to obtain heat transfer properties.

The characteristic curve describes the relation between the specific volume of the adsorbed fluid (*W*) and the adsorption potential (*A*). This curve combines the adsorption values with the temperature-dependent physicochemical properties of the fluids. These are the vapor saturation pressures, which we calculate using the Peng–Robinson equation of state and the density of the fluid in the adsorbed phase, which we obtain using the Hauer model (see Section S3 of the ESI for more details). Using the characteristic curve, we can determine the loading dependence of the specific or differential adsorption enthalpy (Δ*h*) or simply adsorption enthalpy. This magnitude is also referred to as isosteric adsorption enthalpy, differential heat of adsorption, or isosteric heat of adsorption, which is the amount of heat released or required during adsorption/desorption cycles. This is calculated using the vaporization enthalpy of the fluid (Δ*H*_vap_), the adsorption potential (*A*), and the entropy change (Δ*S*). At the same time, the entropy term is expressed as a function of the thermal expansion of the confined fluid (α_ads_), the volumetric uptake (*W*), and the slope of the characteristic curve. Finally, we obtained the thermochemical storage density (SD) of each working fluid from the numerical integration of the specific adsorption enthalpy as a function of the loading within the adsorption and desorption temperature range (see Section S3 of the Supporting Information for specific details).

## Results and Discussion

**Figure 1:**
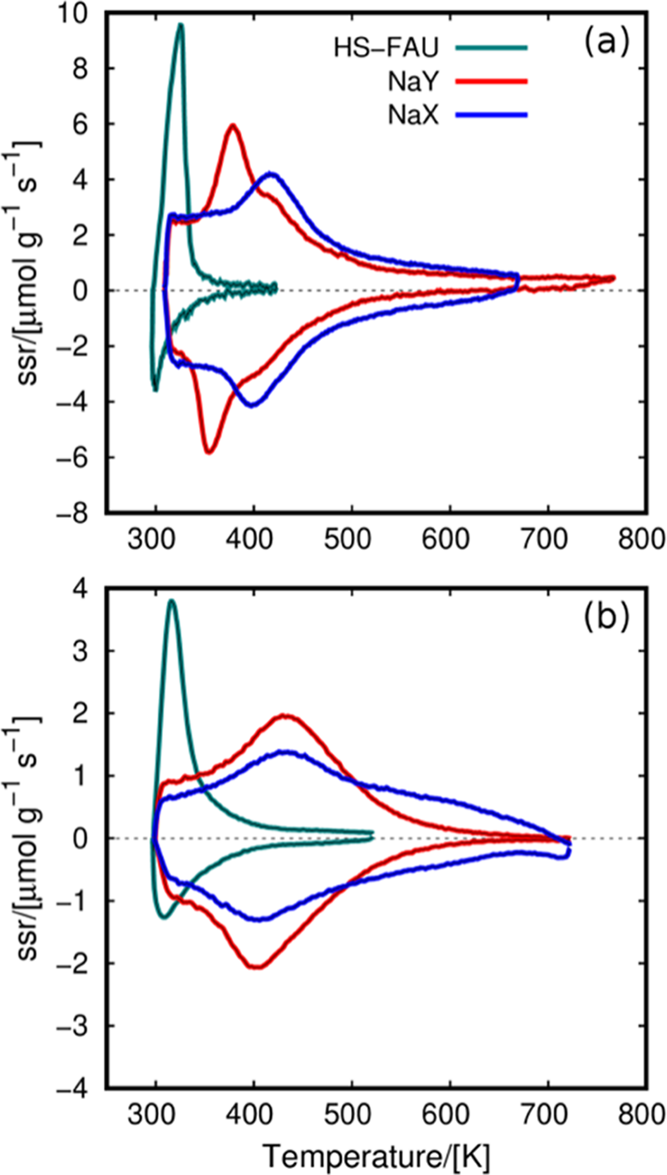
QE-TPDA profiles of (a) water and (b) methanol in FAU zeolites. ssr stands for specific sorption rate, which is proportional to the change of concentration of the adsorptive in the helium stream flowing through the sample. Positive values correspond to desorption branches, while negative values correspond to adsorption cycles. The values of partial pressure for water and methanol in HS-FAU, NaY, and NaX are 1.98, 2.8, and 3.1 kPa, respectively (water), and 0.7 kPa (methanol).

Figure 1 shows the QE-TPDA profiles in the studied faujasites, where the profile above the baseline (ssr = 0) reflects the desorption process and the profile below the baseline reflects the adsorption process. The intensity of the profiles corresponds to the instantaneous concentration of adsorbate desorbed or adsorbed in the material at a given temperature.

The profiles reveal differences in the adsorption of water and methanol. For HS-FAU, we found very sharp profiles both for water and methanol. This means that adsorption occurs abruptly in a narrow temperature range. For NaY and NaX, the low-temperature adsorption at 300–350 K corresponds to high-density states where the guest–guest interactions are of great importance. Figure 1a shows that most water is adsorbed in NaY and NaX between 375 and 475 K. A long tail at higher temperatures is most likely due to the interactions of the water molecules with the cations. This effect is more pronounced for NaX, which has more cations than NaY. The profiles for methanol (Figure 1b) are similar than for water. Desorption maxima and adsorption minima for NaY and NaX are shifted toward higher temperatures than for water, up to ca. 530 K. Also, the broad high-temperature tail for NaX is extraordinarily intensive. Generally, the QE-TPDA profiles show that adsorption is stronger for methanol than for water in NaY and NaX. The interactions between methanol and NaX cations are particularly strong.

**Figure 2:**
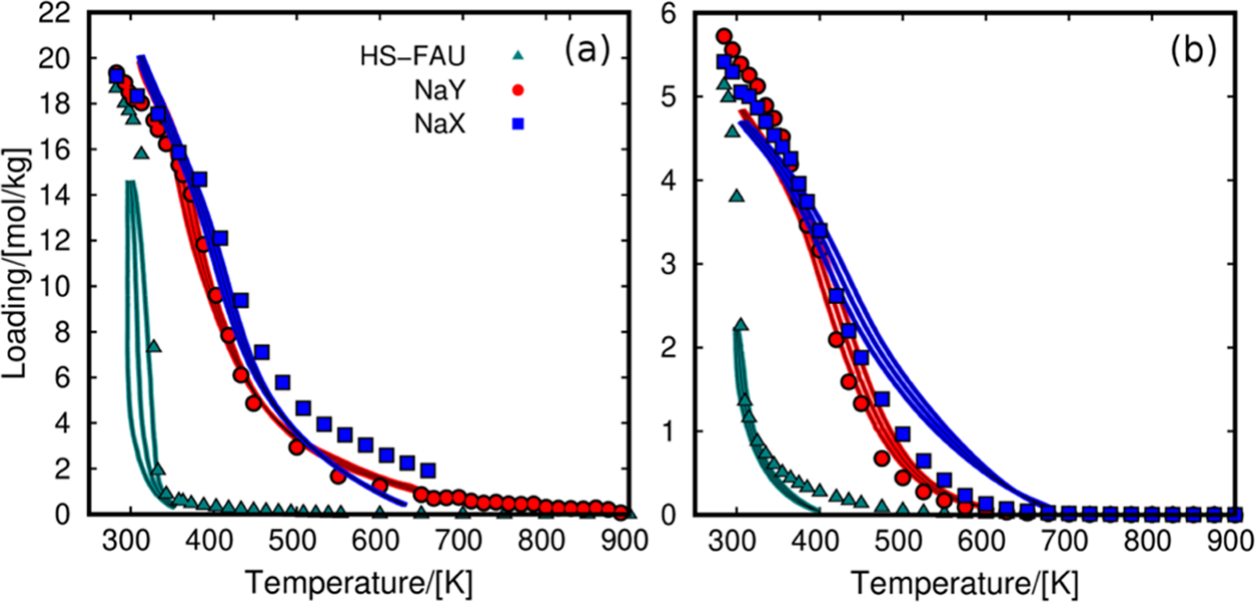
Experimental (lines) and calculated (symbols) adsorption isobars for (a) water and (b) methanol in FAU zeolites. Each experimental isobar is divided into three curves corresponding to adsorption and desorption cycles (obtained from the QE-TPDA profiles) and the average of them. The values of partial pressure for water and methanol in HS-FAU, NaY, and NaX are 1.98, 2.8, and 3.1 kPa, respectively (water), and 0.7 kPa (methanol).

The adsorption isobars can be obtained by integrating the QE-TPDA profiles.^63^ We used the adsorption isobars of methanol for the parameterization of the force field required for molecular simulation (Table S2). Figure 2 compares the experimental and computed adsorption isobars under the same working conditions (see Figure 1). Considering that we are using the same set of (transferable) parameters and partial charges for all of the systems, we found good agreement with the experimental results.

The behavior of the adsorption isobars is similar for water and methanol, since both fluids are polar. The hydrophobicity of HS-FAU, due to the low content of Na cations, leads to a steeped isobar at low values of temperature. While increasing the cations content in the zeolites, the shape of the isobar shows a smooth loading decrease reaching desorption temperatures at about 600 K. This proves the high affinity of polar fluids for the extra-framework cations of the zeolites. The adsorption of methanol in the Na-FAU zeolites shows a minor hysteresis loop, a displacement between adsorption and desorption. Similarly, the adsorption isobar of water in HS-FAU shows a tiny hysteresis loop, which is lower for the zeolites with higher cation content. The set of parameters was then fitted to the intermediate curve, which is the average of adsorption/desorption from experimental measurements (Figure 2).

**Figure 3:**
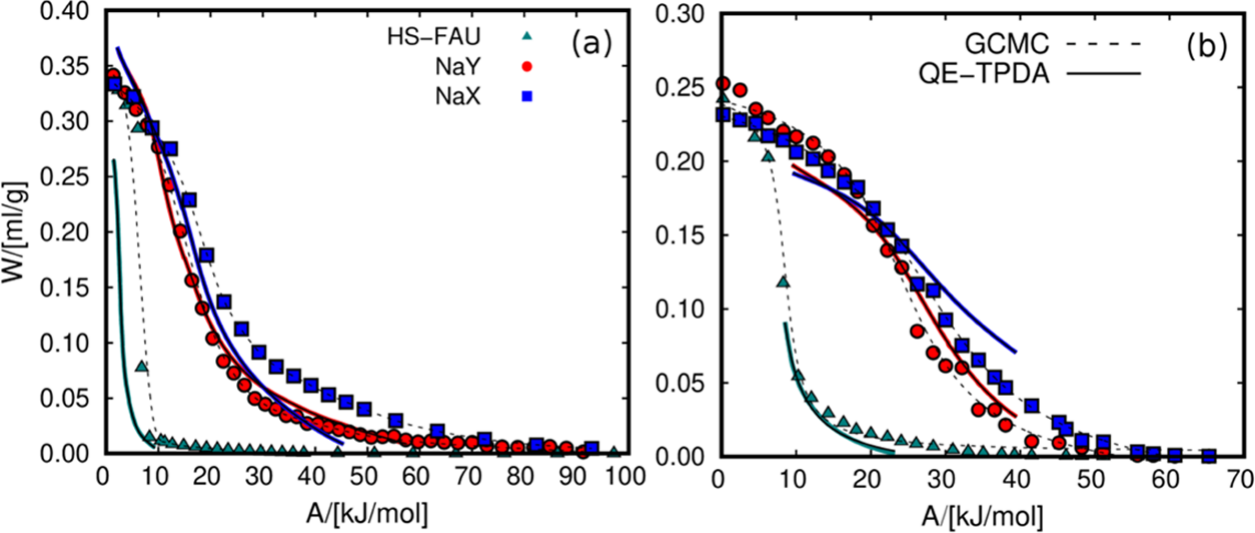
Characteristic curves of (a) water and (b) methanol in FAU zeolites using data from QE-TPDA (solid lines) and CGMC (dashed lines). The dashed lines represent the fitted curve using splines. *W* represents the volume of fluid adsorbed in the micropores, and *A* is the adsorption potential.

Since partial pressures of methanol and water adsorption are different, it is not possible to directly compare saturation loadings of the two fluids from the adsorption isobars. However, we can convert each adsorption isobar to its corresponding characteristic curve, which only depends on the fluid-zeolite working pairs. Figure 3 shows the characteristic curves of water and methanol obtained from the adsorption data. For the GCMC curve, we used the adsorption isobar from Figure 2 and additional adsorption isotherms (Figure S2) to complete the characteristic curve, ranging from zero-coverage to saturation conditions. The data from independent adsorption isobars and isotherms fall into the same characteristic curve. We found that the volumetric adsorption is considerably higher for water (about 0.35 mL of fluid per gram of adsorbent) than for methanol (0.25 mL/g). This is due to the smaller size of water that can connect through four hydrogen bonds per molecule.^83^ Methanol can connect through two,^84,85^ leading to a worse molecular packaging. Another relevant factor for the higher adsorption of water compared to methanol is that, contrary to methanol,^86^ the water molecules can enter the small sodalite cages of FAU zeolites.^87^ For this reason, the free volume for the adsorbents is larger for water than for methanol. To increase the limited number of points obtained from the GCMC simulation, we use splines. It is important to use smooth functions that fit the data well to minimize the noise in the calculations involving the characteristic curves. The fitting for the experimental characteristic curve is more straightforward since it contains more points resulting from the measurements for small temperature increments. The characteristic curves were complemented with adsorption at high temperatures to reach the low-coverage regime.

The presence of cations in the FAU zeolites does not alter their pore volume significantly.^59^ This is why adsorption isobars and characteristic curves have similar saturation values, independently of the cation content. However, the concentration of cations influences the hydrophilic/hydrophobic nature of the zeolites. The adsorption trend of both fluids in NaY and NaX is very similar despite the differences in the number of cations. The curves in NaY are slightly shifted to lower values of temperature (Figure 2) or lower adsorption potential (Figure 3). This effect is not as strong as for the n-alkanes.^59^ As discussed earlier, Ristić et al.^48^ proposed a post-synthesis chemical treatment of NaY to control the desorption temperatures of water. However, all modified samples contained a similar Si/Al ratio, and they found a decrease of the desorption temperatures of about 30 K compared to the original NaY zeolite. To control the desorption temperatures over a wider range of working conditions, we suggest to reduce the cation content to values between HS-FAU (Si/Al ratio = 100) and NaY (Si/Al ratio = 2.61).

**Figure 4:**
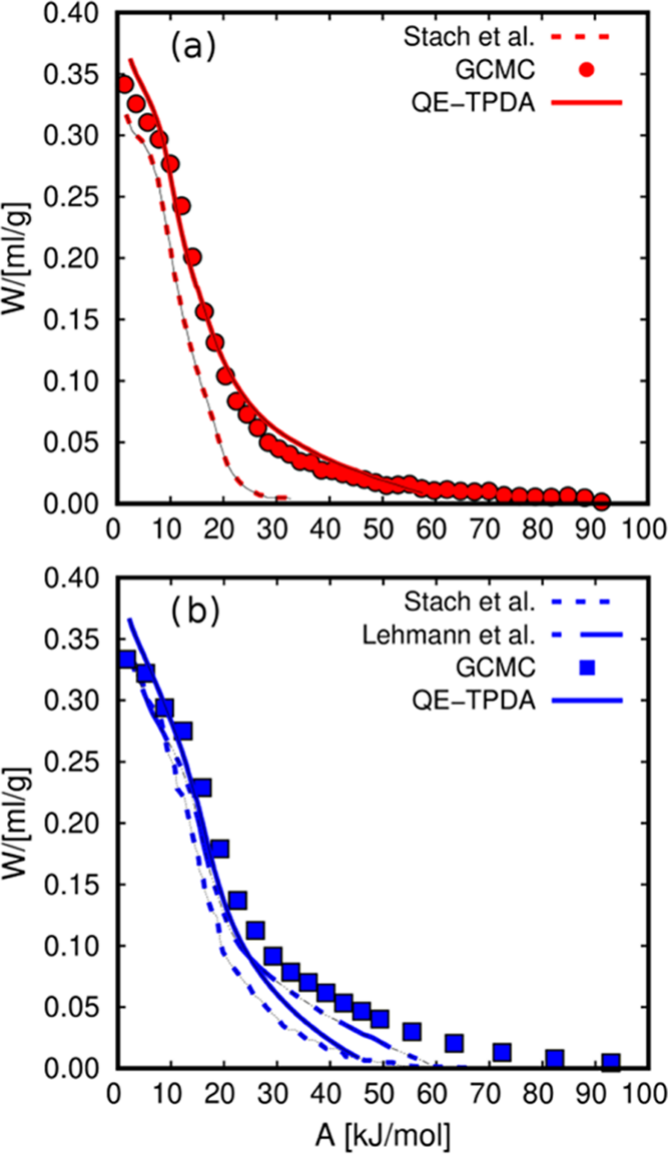
Characteristic curves of water in (a) NaY and (b) NaX obtained from GCMC (symbols) and experiments (lines). The experimental values are from QE-TPDA and reported by Stach et al.^54^ and Lehmann et al.^53^*W* represents the volume of fluid adsorbed in the micropores, and *A* is the adsorption potential.

Reported adsorption studies for heat storage applications typically measure adsorption isotherms at a wide range of temperatures. Then, the adsorption isotherms are reduced to a common characteristic curve. Instead of doing this, here we measured and computed a single adsorption isobar to obtain the temperature dependence of the loading needed for further calculations of the storage density. Then, if necessary, we completed the low-coverage regime of the computed curve with data from additional adsorption isotherms. To validate this approach, we compared the characteristic curves for water obtained in this work from QE-TPDA and GCMC simulation with those reported by Lehmann et al.^53^ and Stach et al.^54^ (Figure 4). Our results are in line with those reported in the literature, with slight deviations mainly due to the use of different commercial samples. In all cases, we observe that the saturation loading (corresponding to *A* → 0 kJ/mol) converges to similar values, i.e., about 0.35 mL/g, which is the saturation value for water in all FAU zeolites (Figure 3). The results shown in Figures 3 and 4 reveal the invariance of the characteristic curves with the adsorption conditions, thus giving consistency to the use of the DP theory for the working pairs of this work.

**Figure 5:**
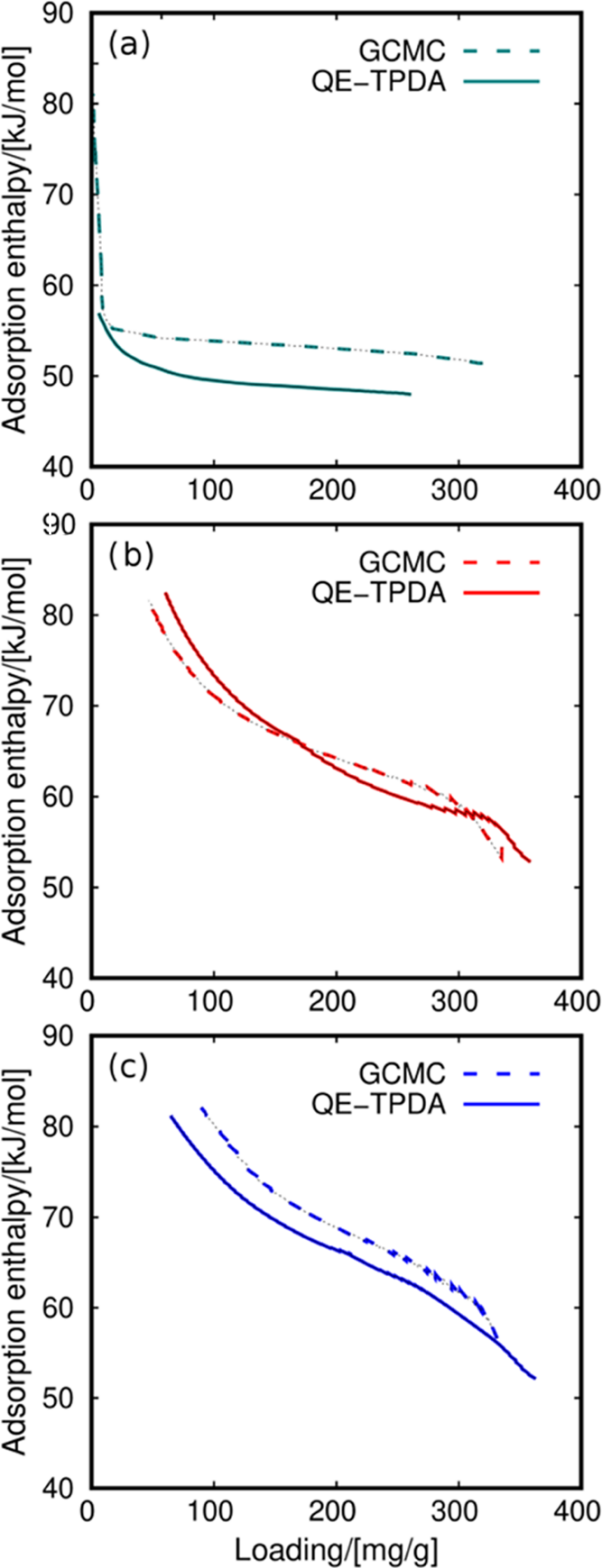
Specific adsorption enthalpy (Δ*h*) of water as a function of loading in (a) HS-FAU, (b) NaY, and (c) NaX. The values were obtained from GCMC simulation (dashed lines) and QE-TPDA (solid lines) data.

**Figure 6:**
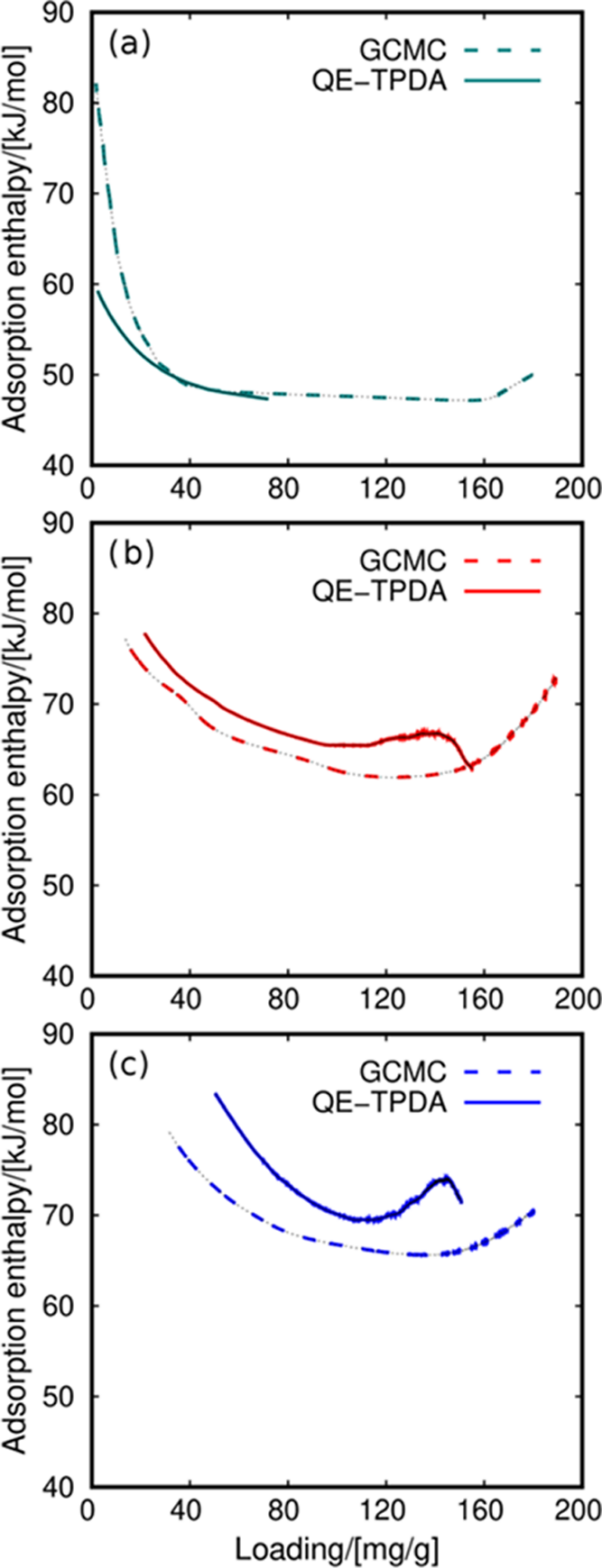
Specific adsorption enthalpy (Δ*h*) for methanol as a function of loading in (a) HS-FAU, (b) NaY, and (c) NaX. The values were obtained from GCMC simulation (dashed lines) and QE-TPDA (solid lines) data.

The performance of a working pair for adsorption-based heat storage depends on two thermodynamical quantities: the adsorption capacity and the adsorption enthalpy at the working conditions. However, these two quantities are not independent, and the adsorption enthalpy can be obtained from the adsorption data and the physicochemical properties of the working fluid. We take the data from the characteristic curves (Figure 3) to obtain the adsorption enthalpy of water and methanol in the three zeolites (Figures 5 and 6) using the DP theory, as described in the Methodology section. The results obtained from the QE-TPDA experiments and GCMC simulation, depicted in Figures 5 and 6, are in agreement, showing similar differences to those found in Figures 2 and 3. Differences between the measured and the computed adsorption isobars shown in Figure 3 entail a deviation of less than 3 kJ/mol in the adsorption enthalpy, except for methanol in NaX, where the difference is about 10 kJ/mol.

The adsorption enthalpy depends on the adsorption behavior and on the physicochemical properties of the working fluid. The properties used in the DP formulation are the vaporization enthalpy, thermal expansion coefficients, liquid density, and saturation pressure. Although Figures 2 and 3 indicate similar behavior for water and methanol adsorption, we found variations in the adsorption enthalpy for these two fluids. These discrepancies are related to the physicochemical properties of water and methanol. Figure 5 shows the loading dependence on the adsorption enthalpy of water in the three zeolites. There is a correlation between the number of cations (degree of hydrophilicity) and the adsorption enthalpy. At low coverage, the absolute value of the adsorption enthalpy is about 80 kJ/mol for the three structures, but the behavior is differentiable at intermediate and higher loadings. For HS-FAU, the adsorption enthalpy shows an abrupt decrease with loading after the low-coverage regime. This phenomenon is related to the low concentration of cations that act as strong interaction centers. Once the first molecules of water are adsorbed near the cations at a low-coverage regime, they quickly nucleate and occupy the rest of the adsorption sites in the structure. Similar findings have been described in the literature^88,89^ for water and methanol adsorption in other sodium-based materials. The decrease in adsorption enthalpy in NaY and NaX is less pronounced than in HS-FAU due to the higher sodium content. Despite the three zeolites having the same FAU topology, they differ in chemical composition, resulting in different strengths and densities of adsorption sites. Thus, the different partial charges on these sites influence the trends in the adsorption enthalpy. At saturation, the adsorption enthalpy is about 50–55 kJ/mol because adsorbate–adsorbate interactions prevail over the interactions with the zeolite.

Figure 6 shows the adsorption enthalpy using methanol as the working fluid. The general trend differs from the values for water shown in Figure 5. The curve corresponding to the adsorption of methanol in HS-FAU is similar to that found for water. The values reach 80 kJ/mol at low coverage and immediately decrease to 50 kJ/mol. However, the sudden decrease of adsorption enthalpy at low coverage is less pronounced for methanol than for water, and the trend shifts slightly at high loading. The most remarkable differences are for NaY and NaX. At low coverage, the values are about 80 kJ/mol, as for the other systems. At intermediate loading, the curves show a minimum at about 60–65 kJ/mol, and the adsorption enthalpy increases to 70–75 kJ/mol at high loading.

**Figure 7:**
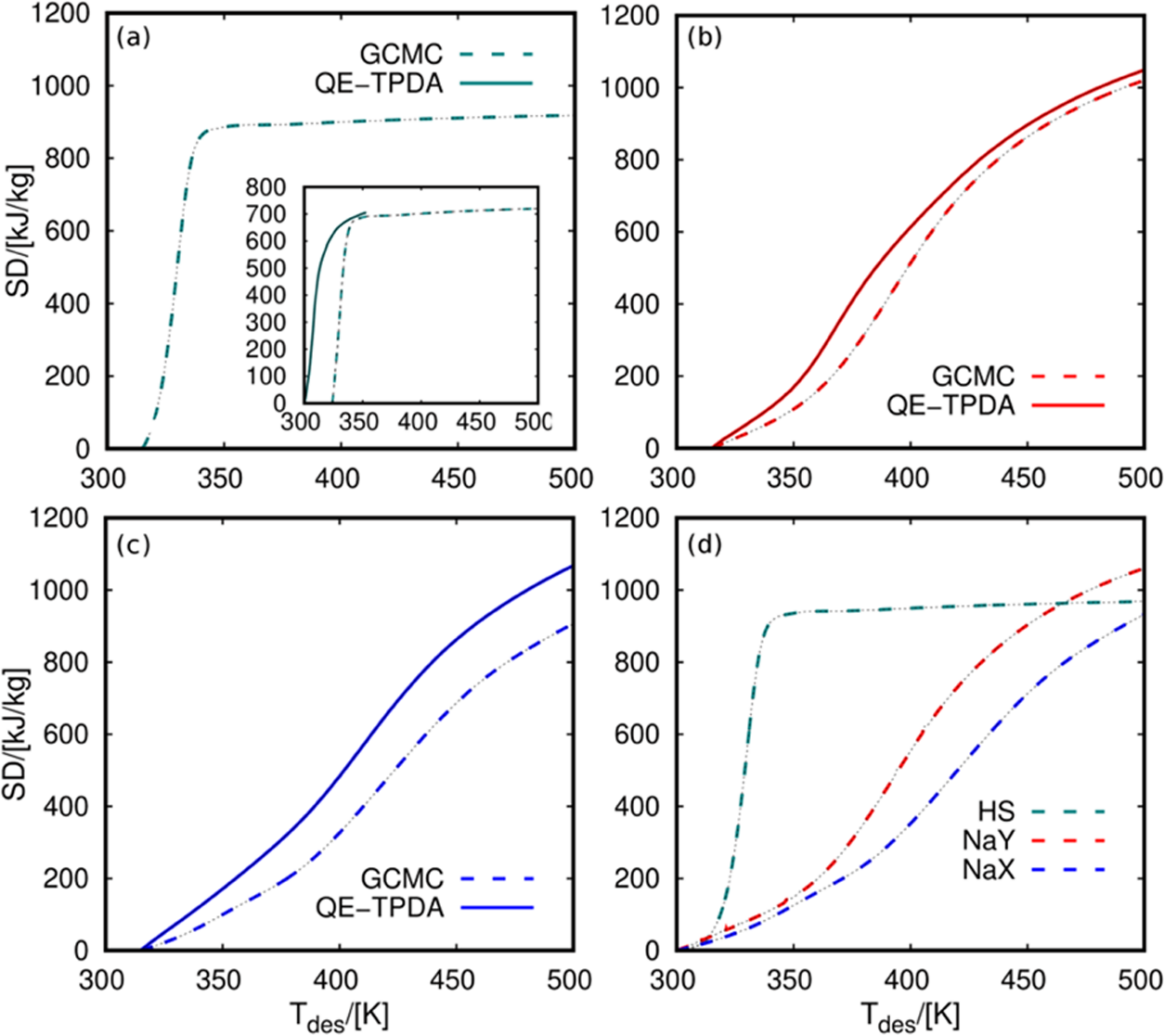
Storage density (SD) of water-zeolite pairs in (a) HS-FAU, (b) NaY, and (c) NaX at *T*_ads_ = 315 K and *P* = 1.98 kPa (HS-FAU), *P* = 2.8 kPa (NaY), and *P* = 3.1 kPa (NaX). The inlet figure in panel (a) is for *T*_ads_ = 300 K (QE-TPDA) and *T*_ads_ = 324 K (GCMC). Panel (d) shows the storage density from GCMC simulation in three zeolites at *T*_ads_ = 300 K. *T*_des_ stands for the temperature of the heat transfer device during the desorption cycles.

One of the main features of an adsorption heat storage device is its energy storage density or simply storage density. We calculated this quantity by integrating the adsorption enthalpy curves between fixed adsorption and desorption temperatures. The values shown in Figure 7a–c were obtained for a fixed adsorption temperature of 315 K. We selected this temperature for being the lowest temperature measured in the QE-TPDA experiments for the three zeolites. The figures show the storage density as a function of the desorption temperature. From these figures, it is possible to compare the values obtained with QE-TPDA and GCMC. These results show similar differences as in previous adsorption isobars (Figure 2) and adsorption enthalpy (Figure 5). However, the operating conditions play an important role here, making comparison more difficult. For example, Figure 7a shows the GCMC values for water in HS-FAU. Because of the high hydrophobicity of this zeolite, the adsorption obtained from QE-TPDA could not reach the saturation capacity of water in HS-FAU. This is because, from the experimental side, establishing adsorption equilibrium in hydrophobic adsorbents takes a long time. The driving force is very low, leading to condensation within the micropores. The inlet figure compares the storage density obtained from QE-TPDA and GCMC using adsorption temperatures of 300 and 324 K, respectively. Using this approximation, the two curves show an analogous abrupt increase, reaching similar storage density values. Extending the GCMC simulations to a wider range of temperatures provides a more detailed analysis of the storage density behavior. Therefore, it could lead to the optimization of the process based on the operational conditions for each working pair. To compare the maximum performance of the three adsorbents, we used the data from GCMC simulations and decreased the adsorption temperature to 300 K. This ensures that all systems reach saturation (Figure 7d). We found two trends. (i) HS-FAU shows an abrupt increase in storage density. The maximum energy is released at relatively low temperatures compared to NaY and NaX because of the rapid desorption in this hydrophobic structure. For example, at a desorption temperature of 350 K, the storage density of HS-FAU surpasses 900 kJ/kg. At the same temperature, NaY and NaX do not even reach 600 kJ/kg. (ii) NaY and NaX have a moderate steep increase, reaching the maximum values at the higher tested desorption temperature, i.e., 500 K. NaY and NaX do not converge to the same storage density value because these zeolites have not released all of the adsorbed water at 500 K (see Figure 2). In contrast, for HS-FAU, the curve is flat at temperature values above 350 K because the zeolite desorbs most of the molecules around this temperature. The three zeolites show a similar maximum value of storage density between 900 and 1100 kJ/kg. It is important to mention that the working pressure of water adsorption in HS-FAU was set to ca. 1 kPa lower than for NaY and NaX (1.98 kPa for HS-FAU, 2.8 kPa for NaY, and 3.1 kPa for NaX). The maximum storage density depends on the maximum loading of water that the adsorbent can capture and release and the exchange adsorption enthalpy. This means that the differences in the maximum storage densities shown in Figure 7d are mainly due to the specific adsorption enthalpy of water (see Figure 5).

It is worth mentioning that the values analyzed above stand for energy per mass of adsorbent; however, the volumetric storage density is another common value analyzed in the literature. Typically, 900–1100 kJ/kg corresponds to 1.4–1.6 GJ/m^3^, which is within the range of the top-performing list of salt hydrates, another common type of thermochemical materials used for heat transfer applications. Donkers et al.^90^ analyzed the thermodynamic data of almost 600 salt hydrates, selecting the 25 top-performing candidates. The salt hydrates of this shortlist exhibit a storage density between 1.6 and 2.7 GJ/m^3^. Hence, water-NaY and water–NaX working pairs show comparable performance with the top-performing salt hydrates for energy storage applications.

**Figure 8:**
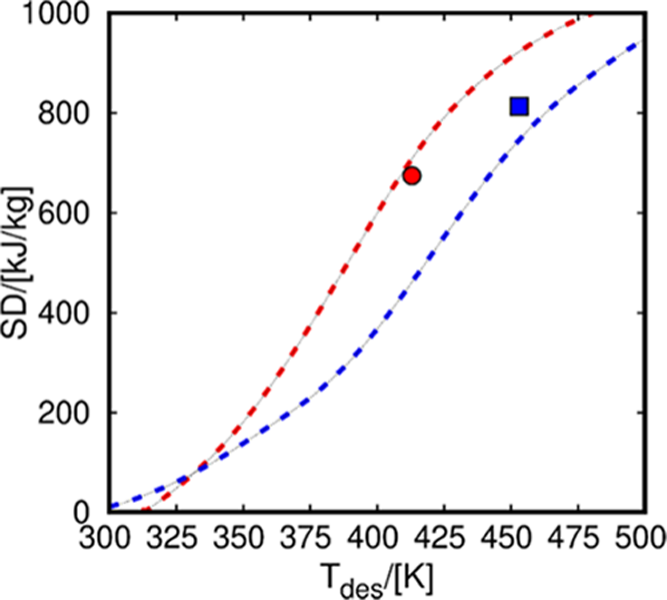
Storage density (SD) of water in NaY (red) and NaX (blue). The values taken from the literature are indicated with symbols.^43,48^ The values resulting from GCMC simulation are in dashed lines. The operational conditions are *T*_ads_ = 313 K and *P* = 1.23 kPa for NaY and *T*_ads_ = 293 K and *P* = 3 kPa for NaX. *T*_des_ stands for the temperature of the heat transfer device during the desorption cycles.

Previous results highlight the importance of the operating conditions to maximize the performance of each fluid-adsorbent working pair. Many works using process simulations or experimental measurements compare the values of several working pairs at single fixed operating conditions. However, the storage density values could change drastically by slightly changing the operating temperature. To compare our approach with the reported data, we computed the storage density of water in NaY and NaX at the same conditions used in previous studies. Ristić et al.^48^ reported a storage density of water in NaY of about 675 kJ/kg (187.5 Wh/kg) for fixed adsorption and desorption temperatures of 313 and 413 K, respectively, and operating pressure of 1.23 kPa. Lehmann et al.^43^ provided storage density values for water in NaX of about 815 kJ/kg (226.38 Wh/kg). However, in this case, the adsorption and desorption temperatures were extended to 293 and 453 K, respectively. In principle, these two values cannot directly be compared, and one could think that NaX shows higher storage densities than NaY. However, extending the desorption temperature, it is possible to analyze the performance of the two systems. In this regard, Figure 8 shows the computed storage density of water in NaY and NaX using reported adsorption conditions as a function of desorption temperature. For comparison, the figure also includes available experimental data. Our predictions are in agreement with the experiments and allow solid comparison between the performance of the two zeolites and water working pairs. To check the effect of the operating pressure on the storage density, Figure S3 shows the results for water using values much lower than the saturation pressure of water. We can observe how the maximum storage density value for each working pair decreases as the pressure decreases up to 0.1 kPa. However, there are no significant differences when using 0.5–1 kPa with respect to the results shown in Figure 7.

**Figure 9:**
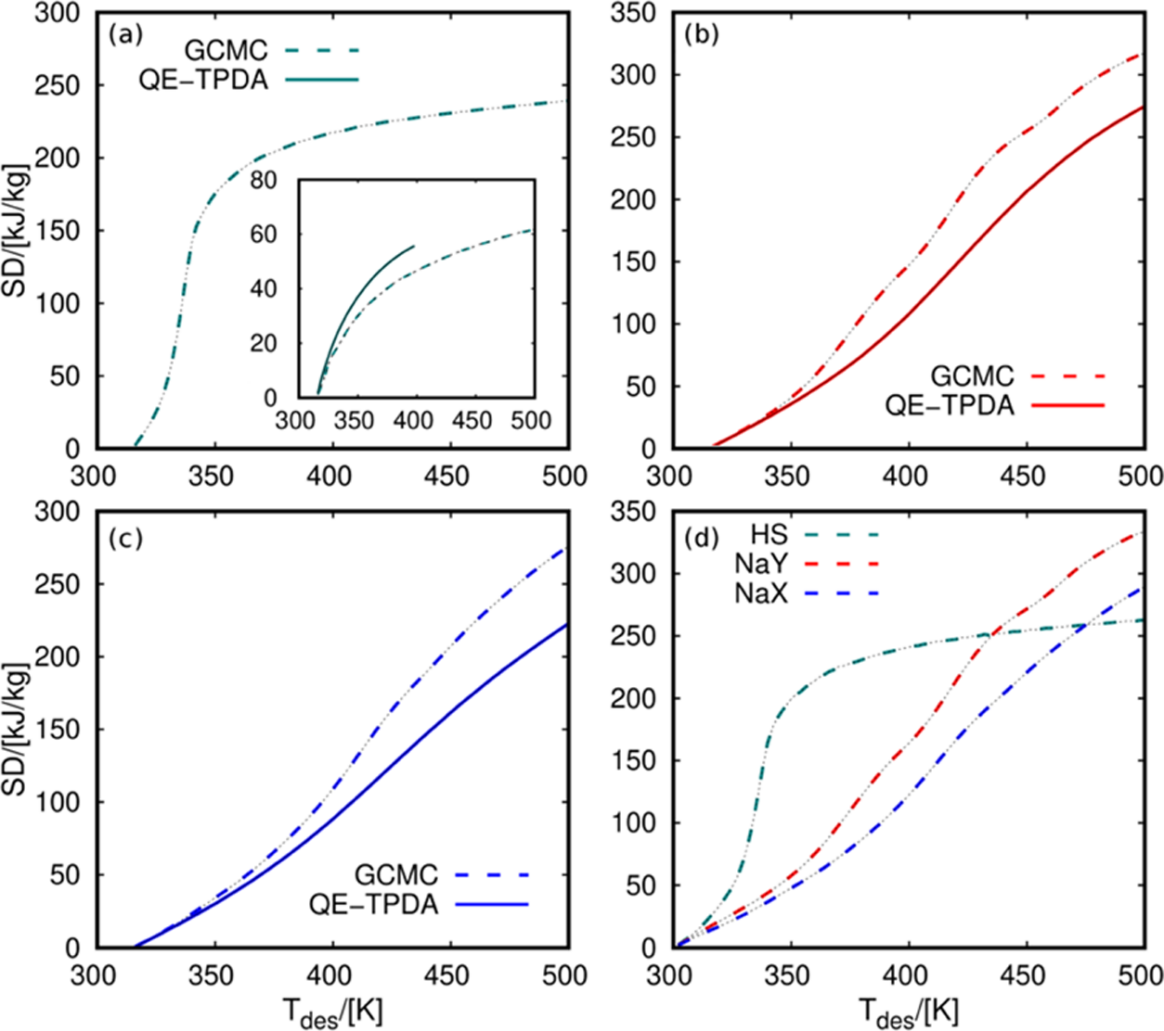
Storage density (SD) of methanol–zeolite pairs in (a) HS-FAU, (b) NaY, and (c) NaX at *T*_ads_ = 315 K and *P* = 5 kPa (HS-FAU) and *P* = 0.7 kPa (NaY and NaX). The inlet figure in panel (a) is for *T*_ads_ = 315 K and *P* = 0.7 kPa. Panel (d) shows the storage density from GCMC simulation at *T*_ads_ = 300 K and *P* = 5 kPa (HS-FAU) and *P* = 0.7 kPa (NaY and NaX). *T*_des_ stands for the temperature of the heat transfer device during the desorption cycles.

To compare the performance of the two working fluids, we calculated the storage density of methanol in the three zeolites using the data obtained with QE-TPDA and GCMC (Figure 9). As for water, the differences between the two techniques are based on the differences found in the adsorption isobars (Figure 2) and the adsorption enthalpy (Figure 5).

The storage densities of methanol depicted in Figure 9 show the same trend as for water (Figure 7), but the maximum values are, on average, between 3 and 4 times lower. Despite the different trends in the adsorption enthalpy of water and methanol (Figures 5 and 6), the absolute values are similar. Another factor that could influence the performance of storage densities when comparing distinct fluids is the vaporization enthalpy. At room temperature, this enthalpy is about 10 kJ/mol higher for water than for methanol. However, the limiting factor comparing the storage densities of the two fluids is the difference in adsorption loading. Figure 2 shows that the FAU zeolites adsorb between 3 and 4 times more water than methanol at low temperatures, which is in line with the trend observed in the storage densities.

## Conclusions

The combination of QE-TPDA experiments with MC simulation gives detailed information on the use of FAU zeolites for heat storage applications. The calculated adsorption isobars show a strong influence of the hydrophobic degree of the adsorbent in the desorption temperatures. HS-FAU desorbs most water and methanol at much lower temperatures than NaY and NaX. This large difference (ca. 200 K) impacts the operating conditions of a heat storage device. Hydrophobic materials such as HS-FAU can be used at low-temperature conditions, *e.g.*, in the 300–350 K range. Simultaneously, hydrophilic adsorbents can operate in high-temperature processes with desorption temperatures over 550 K. This suggests the possibility of tuning the Si/Al ratio to maximize the efficiency of adsorbate–fluid working pairs for given operational conditions.

We calculated characteristic curves, specific adsorption enthalpy, and storage densities of the working pairs using a thermodynamical model based on the theory of adsorption of Dubinin–Polanyi. The choice of the operating conditions for each adsorbent–fluid working pair is crucial. This is a limiting factor for the performance of materials or working fluids. The thermodynamical model provides insights into the performance of a heat storage device by only combining adsorption data with some physicochemical properties of the fluids. These are the density, saturation pressure, and enthalpy of vaporization in a range of operational temperatures and pressures. These properties can be obtained not only from experiments but also from molecular simulations. This could be useful for the screening of adsorbent–fluid working pairs oriented to energy storage applications.

The energy released upon heating and cooling a fluid is higher for water than for methanol. We found storage densities of the water–zeolite pairs to be higher than 1000 kJ/kg, while for methanol–zeolite pairs, they were about 350 kJ/kg. The water/methanol ratio of storage densities is related to the ratio of their adsorption loading. The highest values of water uptake are due to both a strong hydrogen bond network and the access of water to the sodalite cages of the FAU zeolites. The agreement found between experiments and simulation allows the use of GCMC simulation for other operational conditions and provides a comprehensive overview of the performance of the working pairs for energy storage.

## Supporting Information Available

Additional information to support methods and models used in this work, including a set of force field parameters, structural model of zeolites, parameterization of methanol–zeolite interactions, thermodynamical model, and additional adsorption isotherms and storage densities (PDF)
