# Supplementary material for: On the Use of Water and Methanol with Zeolites for Heat Transfer
Source: ACS Sustain Chem Eng. 2023 Mar 8;11(11):4317–28. doi: 10.1021/acssuschemeng.2c05369 (PMC10031555; doi:10.1021/acssuschemeng.2c05369)
Supplement: Supplementary file 1 — sc2c05369_si_001.pdf [file sc2c05369_si_001.pdf]

SUPPORTING INFORMATION

of the paper entitled

On the use of Water and Methanol with Zeolites for Heat Transfer

Rafael M. Madero-Castro[a], Azahara Luna-Triguero[b,c], Andrzej Sławek[d,e], José Manuel Vicent-Luna[f]*, and Sofia Calero[a,f]*

[a]Department of Physical, Chemical, and Natural Systems, Universidad Pablo de Olavide. Ctra. Utrera km. 1. ES-41013 Seville, Spain.
[b]Energy Technology, Department of Mechanical Engineering, Eindhoven University of Technology, P.O. Box 513, 5600 MB Eindhoven, The Netherlands.
[c]Eindhoven Institute for Renewable Energy Systems (EIRES), Eindhoven University of Technology, PO Box 513, Eindhoven 5600 MB, the Netherlands
[d]Academic Centre for Materials and Nanotechnology, AGH University of Science and Technology, Kawiory 30, 30-055 Kraków, Poland.
[e]Faculty of Chemistry, Jagiellonian University, Gronostajowa 2, 30-387 Kraków, Poland.
[f]Materials Simulation and Modelling, Department of Applied Physics, Eindhoven University of Technology, P.O. Box 513, 5600MB Eindhoven, The Netherlands.

Corresponding Authors

* José Manuel Vicent-Luna, e-mail: j.vicent.luna@tue.nl;
* Sofía Calero, e-mail: s.calero@tue.nl

Number of pages: 8 Number of tables: 3 Number of figures: 3

Outline

**Section S1**	**Page S2**
**Section S2**	**Page S2**
**Section S3**	**Page S4**
**Table S1**	**Page S3**
**Table S2**	**Page S3**
**Table S3**	**Page S4**
**Figure S1**	**Page S5**
**Figure S2**	**Page S7**
**Figure S3**	**Page S7**
**References**	**Page S8**

Section S1. Structural model of zeolites.

All zeolites were generated following the same procedure regardless the Si/Al ratio. The unit cell of the pure silica FAU contains 192 Si atoms. We substitute some Si atoms by Al atoms to reproduce the experimental chemical composition (Si/Al ratio of 100, 2.61, and 1.06, HS-FAU, NaY, and NaX, respectively). HS-FAU, NaY, and NaX contain 2, 56, and 88 Al atoms, respectively.

We generated the structures following the methodology described in previous works [1,2]. We started from the crystallographic positions of the pure silica zeolite from the International Zeolite Association (IZA) database [3] to construct the aluminosilicates. For each structure, we created a set of 50 configurations by randomly substituting some silicon atoms by aluminum atoms within the constraint of Löwenstein's rule and selected the most energetically favorable configuration. Then, we compensated the net negative charge of the adsorbents by placing sodium extra-framework cations in the most probable crystallographic positions reported in the literature. A detailed description of these extra-framework cations is given in references [4-6]. Once we added the extra-framework cations to their preferential location, we optimized the structures with energy minimization simulations using Baker's [7] method and a full-flexible core-shell potential.[8,9]

Section S2. Parameterization of methanol-zeolite interactions.

Interactions parameters between the molecules of water and the HS-FAU and NaX zeolites were developed in our previous work [10] using experimental adsorption isobars as reference data. In this work, we also computed the adsorption isobar of water in NaY, showing that the water-zeolite interactions are transferable in the whole range of Si/Al substitutions. Here we followed a similar procedure to obtain the methanol-zeolite interactions. Starting from the cross-term Lennard-Jones parameters for each pseudo atom of the methanol-zeolite pairs, we iteratively modify the ε and σ parameters, creating a matrix of values smaller and larger than the initials. The partial charges for the adsorbates and zeolites are kept fixed and given in Table S1. For each set of parameters, we computed five values of an adsorption isobar from the low to the high coverage regime. We first compare with experimental data for NaX to narrow the search of adequate parameters to reproduce the adsorption in the zeolite with the highest content of extra-framework cations. Then, we compare with the measured data for HS-FAU and finally for NaY. We repeated the process until we found reasonable agreement between experiments and simulations using the same set of Lennard-Jones parameters regardless the Si/Al ratio. The optimal values are provided in Table S2 and the validation against experimental values is shown in Figure 2 of the manuscript.

Table S1. Lennard-Jones parameters and charges of each pseudo-atom for the adsorbent and adsorbates. O_{zeo-Si} and O_{zeo-Al} are the oxygen atoms bridging to silicon and aluminum atoms, respectively.

$$V_{ij} = 4\varepsilon_{ij}\left(\left(\frac{\sigma_{ij}}{r_{ij}}\right)^{12} - \left(\frac{\sigma_{ij}}{r_{ij}}\right)^{6}\right)$$

Molecule	Atom	q[e]	ε/k_B [K]	σ[Å]
**water**[a]	O_w	-0.8476	78.2878	3.165
	H_w	0.4238	0*	0*
**methanol**[b]	CH_3	0.265	98	3.75
	O_{alc}	-0.7	93	3.02
	H_{alc}	0.435	0*	0*
**zeolite**[c]	Si_{zeo}	2.05	22	2.3
	O_{zeo-Si}	-1.025	53	3.3
	Al_{zeo}	1.75	22	2.3
	O_{zeo-Al}	-1.2	53	3.3
	Na	1	0*	0*

*"0" indicates this atom does not interact through van der Waals potential.

[a] reported in reference [11], [b] reported in reference [12], and [c] reported in reference [13],

Table S2. Lennard-Jones parameters to describe the interactions between the zeolite and the water and methanol molecules.

Molecule	Pair interaction	ε/k_B[K]	σ[Å]
**water** [a]	O_w-O_{zeol}	80	3.3
	O_w-Na^+	50	3.3
**methanol** [b]	CH_3-O_{zeol}	80.0	3.8
	O_{alc}-O_{zeol}	70.0	3.8
	CH_3-Na^+	80.0	3.6
	O_{alc}-Na^+	80.0	3.2

[a] Taken from ref [10]. [b] This work.

Table S3. Intramolecular force field for water and methanol molecules.

a) Bonds

$$V_{ij} = \frac{1}{2}k_r(r - r_0)^2$$

Molecule	Atom i	Atom j	r_0[Å]	$k_r/k_B[K]$
**water** [a]	O_w	H_w	1.0	415144.47
**methanol** [b]	CH_3	O_{alc}	1.43	∞*
	O_{alc}	H_{alc}	0.945	∞*

*"∞" indicates a fixed bond

b) Bends

$$V_{ij} = \frac{1}{2}k_\theta(\theta - \theta_0)^2$$

Molecule	Atom i	Atom j	Atom k	θ_0[º]	$k_\theta/k_B[K]$
**water** [a]	H_w	O_w	H_w	109.47	46087.0528
**methanol** [b]	CH_3	O_{alc}	H_{alc}	108.5	55400

[a] Bond and bend parameters reported in reference [11] (water) and [b] reported in reference [12] (methanol).

Section S3. Thermodynamical and mathematical model.

We used the mathematical model based on the Dubinin-Polanyi theory [14,15] to obtain the energy storage properties of the zeolite-fluid working pairs. We first convert the adsorption isobars into their corresponding characteristic curves. The characteristic curve relates the volumetric uptake $W(p,T)$ (volume of fluid adsorbed in the micropores [ml/g]) and the adsorption potential $A(p,T)$ [kJ/mol]. The adsorption potential is the molar free energy of adsorption with opposite sign ($A = -\Delta G$).

$$A(p,T) = RT \ln p_{sat}(T)/p \quad \textbf{(1)}$$

$$W(p,T) = q(p,T)/\rho(T) \quad \textbf{(2)}$$

where $p_{sat}(T)$, is the temperature-dependent vapour saturation pressure of the working fluid, $q(p,T)$, the loading of adsorbed fluid per mass of adsorbent, [g/g], and $\rho(T)$, the density of fluid confined within the micropores [g/ml]. We use the Peng Robinson equation of state to calculate the saturation pressure of each fluid.[16] We obtained the loading of fluid from QE-TPDA experiments and GCMC simulation. We used the model of Hauer to obtain the density of

confined fluids within the micropores.[17,18] This model gives a linear relationship between the density of a fluid confined within the pores of an adsorbent and the operational temperature:

$$\rho(T) = \rho_0(T_0) \cdot [1 - \alpha_T(T - T_0)] \quad \textbf{(3)}$$

where ρ_0 is the free liquid density at the reference T_0 (283.15 K for water [15] and 298 K for methanol [19]). α_T is the free liquid thermal expansion coefficient of each working fluid at the reference temperature and 100 MPa [18,19] ($3.871 \cdot 10^{-4}$ K^{-1} for water and $8.026 \cdot 10^{-4}$ K^{-1} for methanol).

One of the properties of interest for an adsorption-based heat storage application is the thermochemical storage density or simply storage density (*SD*). For a given pressure, the *SD* can be obtained by integrating the loading dependence of the specific enthalpy curves within two selected adsorption and desorption temperatures (Figure S1):

$$SD = \int_{q(T_{ads})}^{q(T_{des})} \Delta h(q) dq \quad \textbf{(4)}$$

where the relation between loading and temperature (adsorption isobars) can be obtained from the characteristic curve and Δh is the specific adsorption enthalpy. The specific adsorption enthalpy, also referred as differential adsorption enthalpy, isosteric adsorption enthalpy, differential heat of adsorption, or isosteric heat of adsorption, is the amount of heat released or required during adsorption/desorption cycles. It should be noted that in this context, Δh is a positive value, even the enthalpy change related to adsorption processes is defined as a negative value.

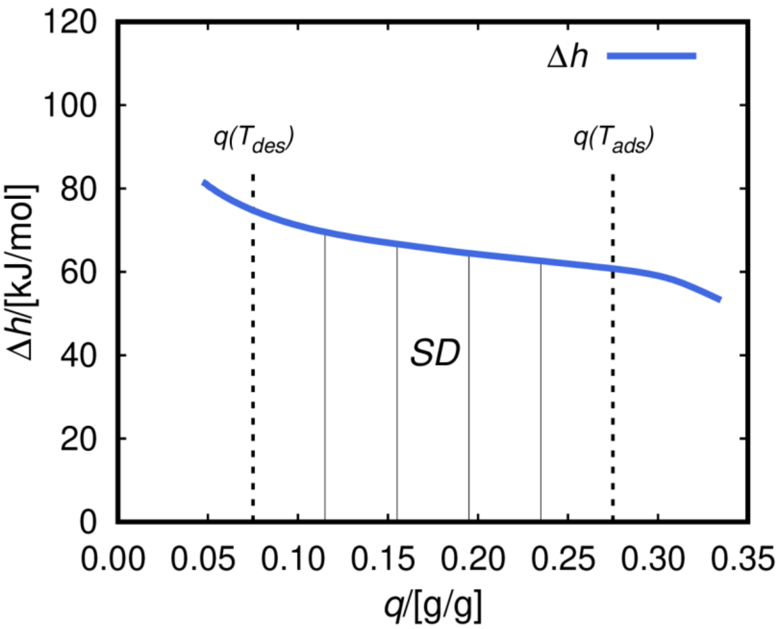

Figure S1. Schematics of storage density calculation (*SD*) from the integration of the specific adsorption enthalpy over adsorption-desorption cycles.

The Dubinin-Polanyi theory also allows determining the specific adsorption enthalpy Δh, which is defined as [19-24]:

$$\Delta h = \Delta H_{vap} + A - T\Delta S \quad \textbf{(5)}$$

where ΔH_{vap} is the enthalpy of vaporization, A is the adsorption potential and ΔS is the differential entropy variation. As mentioned above, Δh is a positive quantity, as well as the three terms in eq. 5. The enthalpy of vaporization ΔH_{vap} is the energy change to transform a substance in liquid phase to gas phase, which is a positive value. This term can be also found in the literature as heat of condensation, usually, when the adsorption enthalpy is referred as heat of adsorption. It should be noted, that in eq. 5, enthalpy of vaporization (or heat of condensation), which depends on the temperature, should express a positive magnitude. The adsorption potential A, is a positive quantity for pressure values below the vapour saturation pressure, and the entropy change ΔS for an adsorption process is negative. The two latter terms in eq. 5 account for the total adsorption enthalpy changes during adsorption processes, which can be related with the adsorption potential (molar free energy, ΔG) as:

$$\Delta G_{ads} = \Delta H_{ads} - T\Delta S_{ads} \ = -A \quad \textbf{(6)}$$

It should be noted that enthalpy is a magnitude that does not depend on the entropy change. As can be inferred from eq. 6, the entropic term in eq. 5 and its negative sign are introduced to cancel the entropic contribution of the molar Gibbs free energy. Finally, the entropy variation [25] is related with the slope of the characteristic curve as:

$$\Delta S = \alpha_{\mathrm{ads}} W \left.\frac{\partial A}{\partial W}\right|_T \quad \textbf{(7)}$$

where α_{ads} is the thermal expansion coefficient of the fluid in the adsorbed phase, obtained from the density model.

In summary, the mathematical model based on the Dubinin-Polanyi theory allows obtaining the storages densities of adsorbent-fluid working pairs, just from an adsorption isotherm or isobar and some physicochemical properties of the fluids. These properties are the enthalpy of vaporization, bulk liquid density, thermal expansion coefficient, and saturation pressure.

Another advantage of the characteristic curve is that it can be reverted to obtain the adsorption isobars or isotherms at different conditions. In addition to the adsorption isobars, we also computed the adsorption isotherms to check the validity of the Dubinin-Polanyi theory. Figure S2 shows the adsorption isotherms of both working fluids in the three zeolites.

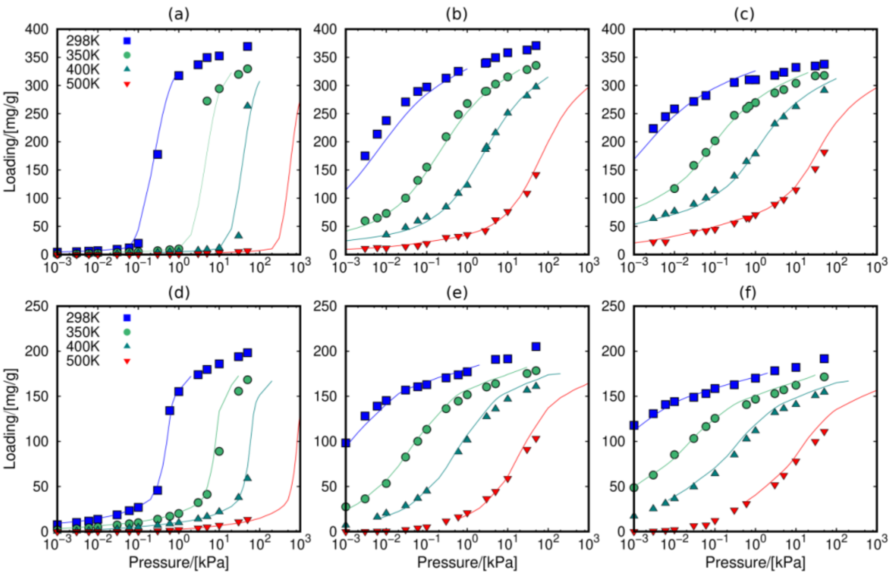

Figure S2. Adsorption isotherms at 298, 350, 400, and 500 K of water (top) and methanol (bottom) in HS-FAU (a, d), NaY (b, e), and NaX (c, f), respectively. Closed symbols represent computed values with GCMC simulations and lines are predicted isotherms obtained from the characteristic curves using the thermodynamical model of Dubinin-Polanyi.

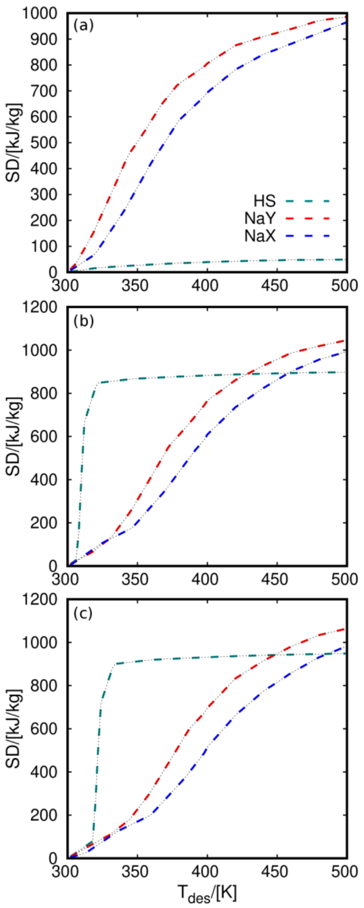

Figure S3. Storage density (SD) of water-zeolite pairs in HS-FAU, NaY, and NaX at Tads=300 K and P=0.1 kPa (a), P=0.5 kPa (b), and P=1 kPa (c).

References:

[1] S.R.G. Balestra, S. Hamad, A.R. Ruiz-Salvador, V. Domínguez-García, P.J. Merkling, D. Dubbeldam, S. Calero, Understanding nanopore window distortions in the reversible molecular valve zeolite RHO, Chem. Mater. 27 (2015) 5657–5667.

[2] A. Luna-Triguero, A. Slawek, R. Sánchez-de-Armas, J.J. Gutiérrez-Sevillano, C.O. Ania, J.B. Parra, J.M. Vicent-Luna, S. Calero, π-Complexation for olefin-paraffin separation using aluminosilicates, Chem. Eng. J. 1224482 (2020).

[3] IZA Database. https://doi.org/10.1016/B978-0-444-53064-6.X5186-X.

[4] D.H. Olson, The crystal structure of dehydrated NaX, Zeolites 15 (1995) 439–443.

[5] G. Engelhardt, Cation location in dehydrated zeolite NaY revisited: SI position is displaced from the center of the hexagonal prism, Microporous Mater. 12 (1997) 369–373.

[6] K. Noe, Crystal structure of zeolite Y as a function of ion exchange, Int. Philos. Q. 55 (2015) 417–436.

[7] Baker, J. An algorithm for the location of transition states. J. Comput. Chem. 7, 385–395 (1986).

[8] Sanders, M. J., Leslie, M. & Catlow, C. R. A. Interatomic potentials for SiO2. J. Chem. Soc. Chem. Commun. 1271–1273 (1984) doi:10.1039/c39840001271.

[9] Jackson, R. A. & Catlow, C. R. A. Computer simulation studies of zeolite structure. Mol. Simul. 1, 207–224 (1989).

[10] Luna-Triguero, A.; Sławek, A.; Huinink, H. P.; Vlugt, T. J. H.; Poursaeidesfahani, A.; Vicent-Luna, J. M.; Calero, S. Enhancing the Water Capacity in Zr-Based Metal-Organic Framework for Heat Pump and Atmospheric Water Generator Applications. ACS Applied Nano Materials 2019, 2 (5), 3050–3059.

[11] Mark, P.; Nilsson, L. Structure and Dynamics of the TIP3P, SPC, and SPC/E Water Models at 298 K. Journal of Physical Chemistry A 2001, 105 (43), 9954–9960.

[12] Stubbs, J. M.; Potoff, J. J.; Siepmann, J. I. Transferable Potentials for Phase Equilibria. 6. United-Atom Description for Ethers, Glycols, Ketones, and Aldehydes. Journal of Physical Chemistry B 2004, 108 (45), 17596–17605.

[13] Calero, S.; Dubbeldam, D.; Krishna, R.; Smit, B.; Vlugt, T. J. H.; Denayer, J. F. M.; Martens, J. A.; Maesen, T. L. M. Understanding the Role of Sodium During Adsorption: A Force Field for Alkanes in Sodium-Exchanged Faujasites. J Am Chem Soc 2004, 126 (36), 11377–11386.

[14] Dubinin, M. M. Theory of the Physical Adsorption of Gases and Vapors and Adsorption Properties of Adsorbents of Various Natures and Porous Structures. Russ Chem Bull 1960, 9, 11072–11078.

[15] Lehmann, C.; Beckert, S.; Gläser, R.; Kolditz, O.; Nagel, T. Assessment of Adsorbate Density Models for Numerical Simulations of Zeolite-Based Heat Storage Applications. Applied Energy 2017, 185, 1965–1970.

[16] Peng, D. Y.; Robinson, D. B. A New Two-Constant Equation of State. Industrial and Engineering Chemistry Fundamentals 1976, 15 (1), 59–64.

[17] Nagel, T.; Beckert, S.; Böttcher, N.; Gläser, R.; Kolditz, O. The Impact of Adsorbate Density Models on the Simulation of Water Sorption on Nanoporous Materials for Heat Storage. Energy Procedia 2015, 75, 2106–2112.

[18] Hauer, A. Beurteilung Fester Adsorbentien in Offenen Sorptionssystemen Für Energetische Anwendungen, 2002. https://doi.org/10.14279/depositonce-469.

[19] Madero-Castro, R. M.; Vicent-Luna, J. M.; Calero, S. Adsorption of Linear Alcohols in Amorphous Activated Carbons: Implications for Energy Storage Applications. ACS Sustainable Chem. Eng., 10, 2022 6509-6520.

[20] C. Lehmann, S. Beckert, R. Gläser, O. Kolditz, T. Nagel, Assessment of Adsorbate Density Models for Numerical Simulations of Zeolite-Based Heat Storage Applications, Appl Energy. 185 (2017) 1965–1970.

[21] A. Ristić, F. Fischer, A. Hauer, N. Zabukovec Logar, Improved Performance of Binder-Free Zeolite Y for Low-Temperature Sorption Seat Storage, J Mater Chem A Mater. 6 (2018) 11521–11530

[22] T. Kohler, K. Müller, Influence of Different Adsorbates on the Efficiency of Thermochemical Energy Storage, Energy Sci Eng. 5 (2017) 21–29

[23] C. Lehmann, S. Beckert, T. Nonnen, J. Möllmer, R. Gläser, O. Kolditz, T. Nagel, A Comparison of Heat Storage Densities of Zeolite Granulates Predicted by the Dubinin-polanyi Theory to Experimental Measurements, Energy Procedia. 105 (2017) 4334–4339.

[24] H. Stach, J. Mugele, J. Jänchen, E. Weiler, Influence of Cycle Temperatures on the Thermochemical Heat Storage Densities in the Systems Water/Microporous and Water/Mesoporous Adsorbents, Adsorption. 11 (2005) 393–404.

[25] Bering, B. P.; Dubinin, M. M.; Serpinsky, V. v. Theory of Volume Filling for Vapor Adsorption. Journal of Colloid and Interface Science 1966, 21, 378–393.
